# Prevalence and levels of disability post road traffic orthopaedic injuries in Rwanda

**DOI:** 10.4102/ajod.v13i0.1251

**Published:** 2024-01-18

**Authors:** JC Allen Ingabire, Aimee Stewart, Jean Baptiste Sagahutu, Gerard Urimubenshi, Georges Bucyibaruta, Sonti Pilusa, Carine Uwakunda, Didace Mugisha, Leontine Ingabire, David Tumusiime

**Affiliations:** 1 Department of Surgery, College of Medicine and Health Sciences, University of Rwanda, Kigali, Rwanda; 2 Department of Surgery, University Teaching Hospital of Kigali, Kigali, Rwanda; 3 Department of Physiotherapy, Faculty of Health Sciences, University of the Witwatersrand, Johannesburg, South Africa; 4 Department of Physiotherapy, College of Medicine and Health Sciences, University of Rwanda,Kigali, Rwanda; 5 Department of Epidemiology and Biostatistics, Faculty of Medicine, Imperial College London, United Kingdom; 6 Department of Surgery, Kibagabaga Level II Teaching Hospital, Kigali, Rwanda; 7 Department of Environmental, College of Medicine and Health Sciences, University of Rwanda, Kigali, Rwanda; 8 Department of Nursing, College of Medicine and Health Sciences, University of Rwanda, Kigali, Rwanda

**Keywords:** prevalence, disability, road traffic injuries, rehabilitation, WHODAS 2.0

## Abstract

**Background:**

Prolonged disability resulting from road traffic injuries (RTIs) contributes significantly to morbidity and disease burden. A good understanding of the prevalence and the level of disability of orthopaedic injuries in developing countries is crucial for improvement; however, such data are currently lacking in Rwanda.

**Objectives:**

To determine the prevalence and levels of disability of 2 years post-road traffic orthopaedic injuries in Rwanda.

**Method:**

A multicentre, cross-sectional study from five Rwandan referral hospitals of 368 adult RTI victims’ sustained from accidents in 2019. Between 02 June 2022, and 31 August 2022, two years after the injury, participants completed the World Health Organization Disability Assessment Schedule (WHODAS 2.0) Questionnaire for the degree of impairment and the Upper Extremity Functional Scale and Lower-Extremity Functional Scale forms for limb functional evaluation. Descriptive, inferential statistics Chi-square and multinomial regression models were analysed using R Studio.

**Results:**

The study’s mean age of the RTOI victims was 37.5 (±11.26) years, with a sex ratio M: F:3: 1. The prevalence of disability following road traffic orthopedic injury (RTOI) after 2 years was 36.14%, with victims having WHODAS score > 25.0% and 36.31% were still unable to return to their usual activities. Age group, Severe Kampala Trauma Score and lack of rehabilitation contributed to disability. The most affected WHODAS domains were participation in society (33%) and life activities (28%).

**Conclusion:**

The prevalence and levels of disability because of RTOI in Rwanda are high, with mobility and participation in life being more affected than other WHODAS domains. Middle-aged and socio-economically underprivileged persons are the most affected.

**Contribution:**

This study showed that a good rehabilitation approach and economic support for the RTI victims would decrease their disabilities in Rwanda.

## Background

Long-term disability post road traffic injury (RTI) is a public health problem that devastates individuals, and has an enormous societal and economic impact in many countries (Gathecha et al. 2018; World Health Organization 2018). Each year 50 million people are injured, 1.2 million worldwide die from RTIs, 30% live with a permanent disability, and 14% are unable to return to work (Alemany, Ayuso & Guillén 2013; Hyder, Puvanachandra & Allen 2013; Oluwaseyi & Gbadamosi 2017). Limb injuries following road traffic crashes are more frequent than other injuries ranging from 36% to 48% (Chichom-Mefire et al. 2018). The International Classification of Functioning, Disability and Health (ICF) Framework defines disability as ‘difficulty in functioning at the body, person, or societal levels, in one or more life domains, as experienced by an individual with a health condition in interaction with contextual factors’ (Perenboom & Chorus 2003). The World Health Organization Disability and Health Assessment Schedule (WHODHAS 2.0) is the most widely accepted tool designed to evaluate disability from the ICF and comprises 12 or 36 items scored over 100 Svanborg et al. 2022).

Long-term disabilities mainly affect the working-age group in low and middle-income countries (LMICs), impairing individual lives, society, and the economy (Grimm & Treibich 2010; Selassie et al. 2008; Üzümcüoǧlu et al. 2016). The prevalence of RTI is much higher in LMICs with complications than in developed countries (De Jongh et al. 2017; World Health Organization 2018). These complications are mainly physical, social and cognitive, affecting the victims of RTIs, their families, and society (Oluwaseyi & Gbadamosi 2017; Sousa et al. 2016). A house survey carried out in Sierra Leone, Rwanda, Nepal, and Uganda found that more than 38.5% of all RTI victims had disabilities following these injuries, with head and extremity injuries being more predominant (Nabeel et al. 2018). Disabilities following RTIs in developing countries are influenced by the severity of the injury and economic factors in the concerned country, especially for the victims’ families (Gathecha et al. 2018). In many developing countries, the prevalence of road traffic disabilities depends on the country, ranging from 1.2% to 14.0% of all the victims mostly from modest-income families (Glèlè-ahanhanzo et al. 2018; Hoang et al. 2020; Lin et al. 2013; Sousa et al. 2016). Even though the prevalence and pattern of long-term disabilities in LMICs are known in some countries, some are still without data (Banks, Kuper & Polack 2017).

Rwanda is in sub-Saharan Africa with a population of more than 13 million; the capital is Kigali. The Rwanda National Institute of Statistics 2022 census estimated that the prevalence of disability in Rwanda was 5%, comprising congenital, genocide against the Tutsi victims, and other disabilities, including RTIs National Institute of Statistics of Rwanda. (2023). In 2019, the Rwanda National Police reported 4661 RTIs, with 700 fatalities. Still, no data exist on the prevalence and level of disability of long-term disabilities attributed to RTI. Studies have shown that better outcomes for these injuries depend on medical care, rehabilitation, and social and economic support, which are still challenging in many LMICs (Grimm & Treibich 2010). Inadequate immediate and rehabilitative care post-injury negatively impacts the victims’ functional outcome and social reintegration and is a challenge in LMICs (Faux et al. 2015; Neagu 2020; Üzümcüoǧlu et al. 2016). In Rwanda, there are many RTI victims (Nabeel et al. 2018), a low number of rehabilitation centres, and insufficient rehabilitation personnel at health facilities affecting the outcome of the victims (Locke et al. 2020). To develop appropriate interventions and compensations for long-term disabilities post-RTOIs, we must understand the prevalence and the level of disabilities following road traffic orthopaedic injuries (Elrud et al. 2019). This study aims to determine the prevalence and levels of disability of 2 years post RTOI in Rwanda.

## Research methods and design

### Study design and study settings

A multicentre cross-sectional study was undertaken to analyse the hospital-based data on road traffic orthopaedic injuries that occurred in 2019, and were treated at the five Rwandan referral hospitals. These hospitals are referral and teaching hospitals with emergency, orthopaedic, and rehabilitation departments. Data were collected between 02 June 2022 and 31 August 2022 from the Centre Hospitalier Universitaire de Kigali (CHUK), the Rwanda Military Hospital (RMH) and King Faisal Hospital (KFH), all located in Kigali City but which receive patients from across Rwanda. The two other hospitals are: Centre Hospitalier Universitaire (CHUB) in the Southern Province and Ruhengeri Hospital (RH) in the Northern Province.

### Study population and sample size

The study population comprised 2019 RTOI survivors aged 18 years and above admitted to the above-mentioned five hospitals for both upper and lower limbs injuries. We used Krejcie and Morgan’s formula (Fincham & Draugalis 2013) for sample calculation and random sampling for sample size. According to the records of these five hospitals, around 4600 cases post-RTIs with 1986 orthopaedic injuries were admitted during the selected study period. The sample size representative of these RTOI victims was 368. We consulted the hospital records from the emergency departments, outpatients and admission for patients’ demographics and contacts, details of the injury pattern, and the length of stay in the hospital. We excluded participants who were not oriented to time and space, and could not respond to the questionnaire and patients with injuries other than orthopaedic injuries. Those fulfilling the inclusion criteria of being above 18 years and having an orthopaedic traffic injury in 2019 were contacted via telephone for their demographic details and requested to come to the hospital for further evaluation.

### Psychometric properties of the instruments

In this study, we used three instruments Upper Extremity Functional Scale (UEFS), Lower-Extremity Functional Scale (LEFS), and WHODAS 2.0, known as Patients Reported Outcomes.

Using the WHO guidelines of translation (Tietschert et al. 2022), the questionnaires were translated from English to Kinyarwanda by two language experts and back to English by two other language experts to address the cultural and linguistic equivalence. Thereafter, the questionnaires were sent to experts in orthopaedic and rehabilitation for their opinion on the quality of translation, clarity, and suitability for the Rwandan participants.

UEFS was used in the evaluation of the upper extremity functional impairment with 20 questions, which demonstrated excellent psychometric properties, including good internal consistency (Cronbach’s alpha > 0.83) and has been validated in many languages (Pransky et al. 1997). LEFS assesses the subjective functional activity performance of daily living in the lower extremities. It was developed and validated for various lower extremity conditions based on the WHO model of impairment, disability and handicap. The LEFS is expected to accurately measure even minor effects of impaired activity performance experienced by participants with lower extremity musculoskeletal dysfunction and has been validated in the Kinyarwanda language (Tumusiime et al. 2014). UEFS Scale and LEFS are self-reported patient questionnaires containing 20 questions about a person’s ability to perform everyday tasks and grade the severity level. The total score is 80 points for all 20 activities computed to 100, where the minimum score is 0, and the maximum score is 100 – the lower the score, the more significant the disability. The classification of functionality level is 0% to 25% – trace functional, 26% to 50% – very poor, 51% to 75% – poor, 76% to 89% – partial functional, and 90% to 100% – fully functional.

The WHODAS 2.0 is a standard multidimensional questionnaire applicable to measure the level of disability across many conditions and has been validated in several languages. Therefore, this schedule has been translated into 16 languages in 14 countries, and has been reported to have adequate internal consistency, construct and discriminate validity (Svanborg et al. 2022). The patient’s overall disability was evaluated using WHODAS 2.0, an assessment tool developed by the WHO to measure disability and functional impairment under the International Classification of Functioning, Disability and Health (ICF) (Svanborg et al. 2022). The WHODAS 2.0 is a self-reported tool, administered to participants aged 18 years and above. The severity of impairment is determined based on the highest qualifier of body functions and structural components of the ICF (no disability: 0% – 4%, mild: 5% – 24% impairment, moderate: 25% – 49% impairment, severe: 50% – 95% impairment, complete: 96% – 100%).

The Kampala Trauma Score (KTS) (Weeks, Juillard & Monono 2014), which was computed as part of the clinical examination to forecast the patient’s prognosis at the time of admission, was used to assess the extent of the injury. The patient’s age, systolic blood pressure, respiration rate, neurological condition, and existence of significant injuries are added up to determine this score. The KTS was then categorised as mild, moderate, or severe, and used to forecast the patient’s prognosis (Haac et al. 2015).

### Procedure

Among 1986 orthopaedic injuries, we reached out to 1721 on the phone, where some have died, or their phones are out of line. After sampling, participants were invited to the hospital to assess their current status after almost 2 years post-RTOIs. The severity of the injury was evaluated using the KTS, which is classified as mild, moderate and severe. For limb function outcome evaluation, we used the UEFS (Pransky et al. 1997) and LEFS (Binkley et al. 1999).

We asked the participants to consider how much their impairments interfered with their lives in the last 30 days and to answer on a 5-point response scale from 0 to 4 (No difficulty-Extreme difficulty). The data collectors helped the participants to fill in the questionnaire if they could not write. We calculated the average score of each WHODAS 2.0 domain, understanding and communicating, getting around, self-care, getting along with others, life activities, and social participation. The participant’s socioeconomic status (Ubudehe) was collected according to the Rwandan government classification, where Category I includes impoverished and vulnerable citizens. Category II includes citizens who can afford some form of rented or owned accommodation but are not gainfully employed and can only afford to eat once or twice a day. Category III includes citizens who were gainfully employed or employers of labour. Category IV are citizens who are chief executive officers of big businesses, full-time employees with organisations, industries or companies, government employees, owners of shops or markets and owners of commercial transport vehicles or trucks (Sabates-Wheeler et al. 2015).

This study has three outcome variables: the prevalence of long-term disabilities, the severity of the disability, and the level of functionality 2 years after RTOI. The explanatory variables include: demographic data, injury category, length of hospital stay, and type of road user (cyclists, drivers, motorcyclists, passengers, and pedestrians) and return to work and rehabilitation.

### Data management and analyses

Data were collected using the questionnaires, entered into the computer by a Google Form data entry mode, and analysed using the R Studio. We performed a descriptive analysis of the patient-reported outcome measure scale (WHODAS 2.0, UEFS, LEFS). Categorical variables were summarised using counts and percentages, continuous variables with means and standard deviations (SD). We used the Kruskal–Wallis and Wilcoxon–Mann Whitney tests to compare three or more and two independent categories, respectively. To evaluate the association between independent variables and WHODAS 2.0 scores, we constructed a multinomial regression model to assess the odds ratio between WHODAS 2.0 scores. We considered *p* < 0.05 to be statistically significant.

### Ethical considerations

We obtained the ethical approval to conduct the study from the University of Rwanda, College of Medicine and Health Sciences Institutional Review Board (18/CMHS IRB/2022). The Rwanda National Research Committee operating in the Ministry of Health approved this study (NHRC/2022/PROT/014) and the collaboration with the Rwanda Biomedical Center (5535/RBC/2022) injury department. We obtained the local ethical approvals from the five hospitals’ ethics committees; CHUK (EC/CHUK/051/2022), CHUB (REC/UTHB/089/2022), RH (313/RRH/DG/2022), KFH (EC/KFH/015/2022), RMH (RMH IRB/027/2022). All participants signed the consent form before enrolment into the study, and all data were kept confidential for only the study’s purposes.

## Results

### Demographic characteristics of the participants

In total, there was 4661 RTIs in 2019, 1986 patients sustained orthopaedic injuries, and we analysed 368 participants. Of these, 64.5% (238 cases) were recruited from CHUK. The mean age of our participants was 37.5 ± 11.26 years, predominant in the age group of 31–50 years, and age was associated with the WHODAS score (*p* = 0.005). Males were predominant at 74.25% (sex ratio M: F:3: 1), and sex was not associated with the disability level (*p* = 0.478). The prevalence of disability was 35.8% (132/368), with the WHODAS score from 25% – 100% in the total sample size, with 63.2% of no disability (0% – 24%). Only 7.58% had no education level and were found to be associated with the level of disability (*p* = 0.005). Most of our participants resided in Kigali city (46.34%), and the residence is statistically significant towards the disability level (*p* = 0.041). Occupations of the RTOI survivors were also associated with their recovery (0.001); 154 (41.73%) of them were in business, and 107 (29%) were in the informal sector (no fixed job). Most of our participants were in Category III of socioeconomic class 227 (61.52), followed by Category II (33.06%), and 64.66% of injuries involved motorcycles as cause where the socioeconomic class is associated with disability outcome (*p* = 0.005) (Table 1).

**TABLE 1:** Demographics profile of participants versus WHODAS score.

Factors	WHODAS score												
	No disability (0% – 4%) (*n* = 95)			Mild (5% – 24%) (*n* = 141)		Moderate (25% – 49%) (*n* = 81)		Severe (50% – 100%) (*n* = 51)		Test statistics		Total (factors)	
	Mean	*N*	%	*N*	%	*N*	%	*N*	%	*X* ^2^	*p*	*N*	%
**Age (in years)**	37.57 (± 11.26)	-	-	-	-	-	-	-	-	19.627	0.005	-	-
18–30	-	41	40.20	36	35.29	15	14.71	10	9.80	-	-	102	27.7
31–50	-	50	22.12	89	39.38	59	26.11	28	12.39	-	-	226	61.41
> 50	-	4	10.00	16	40.00	7	17.50	13	32.50	-	-	40	10.87
**Sex**	-	-	-	-	-	-	-	-	-	*W* = 12 248	0.478	-	-
Male	-	73	26.64	107	39.05	59	21.53	35	12.77	-	-	274	74.25
Female	-	22	23.40	34	36.17	22	23.40	16	17.02	-	-	95	25.75
**Level of education**	-	-	-	-	-	-	-	-	-	22.334	0.005	-	-
None	-	4	14.81	11	40.74	7	25.93	5	18.52	-	-	28	7.58
Primary	-	32	18.60	72	41.86	40	23.26	28	16.28	-	-	172	41.73
Secondary	-	30	27.27	40	36.36	26	23.64	14	12.73	-	-	110	29.81
University	-	29	49.15	18	30.51	8	13.56	4	6.78	-	-	59	15.99
**Residence**	-	-	-	-	-	-	-	-	-	6.3633	0.041	-	-
Kigali City	-	55	32.16	61	35.67	36	21.05	19	11.11	-	-	46.5	-
Secondary cities	-	22	23.40	37	39.36	17	18.09	18	19.15	-	-	25.5	-
Other Districts	-	18	17.48	43	41.75	28	27.18	14	13.59	-	-	28	-
**Occupation**	-	-	-	-	-	-	-	-	-	20.498	0.001		
Farmer	-	6	19.35	17	54.84	3	9.68	5	16.13	-	-	31	8.40
Business	-	44	28.57	60	38.96	33	21.43	17	11.04	-	-	154	41.73
Students	-	1	20.00	0	0.00	3	60.00	1	20.00	-	-	5	1.36
Public service	-	20	34.48	19	32.76	14	24.14	5	8.62	-	-	58	15.72
Informal sector	-	18	16.98	38	35.85	28	26.42	22	20.75	-	-	107	29
Retired	-	6	42.86	7	50.00	0	0.00	1	7.14	-	-	14	3.79
**Socio-economic status (Ubudehe)**	-	-	-	-	-	-	-	-	-	10.516	0.005	-	-
I	-	3	15.00	4	20.00	6	30.00	7	35.00	-	-	20	20
II	-	21	17.36	56	46.28	23	19.01	21	17.36		-	122	33.06
III	-	71	31.28	81	35.68	52	22.91	23	10.13	-	-	227	61.52
**Cause of the Injury**	-	-	-	-	-	-	-	-	-	7.16	0.066	-	-
Cars	-	30	33.71	20	22.47	20	22.47	19	21.35	-	-	89	24.20
Motorcycles-	-	50	44.25	18	15.93	34	30.09	11	9.73	-	-	113	30.70
Motorcycles-Cars	-	40	32.00	36	28.80	33	26.40	16	12.80	-	-	125	33.96
Others	-	21	51.22	7	17.07	8	19.51	5	12.20	-	-	41	11.14

### Clinical factors

In post-RTOIs, half of our participants were managed within 1 day (49.32%), with a mean of 30 days and 42.01% were treated by Open Reduction and Internal Fixation (ORIF). The majority were discharged within 14 days (40.38%). Our findings show that KTS is associated with the patient’s outcome after the accident (*p* = 0.041), and 246/368 (66.84%) patients had moderate KTS. After injury management, 37.13% of the victims could not undergo any rehabilitation management, and this was associated with the patient outcome (*p* < 0.001) (Table 2). The UEFS score had a mean of 93, and the LEFS score had a mean of 75 in the total sample size. Comparing the LEFS and the level of disability according to WHODAS total score, it is statistically significant (*p* < 0.001)) and also significant for UEFS (*p* = 0.006).

**TABLE 2:** Clinical factors versus WHODAS.

Factors	WHODAS score										Total (factors)	
	No disability (0% – 4%) (*N* = 95)		Mild (5% – 24%) (*N* = 141)		Moderate (25% – 49%) (*N* = 81)		Severe (50% – 100%) (*N* = 51)		Test statistics			
	*N*	%	*N*	%	*N*	%	*N*	%	*X* ^2^	*p*	*N*	%
**Kampala Trauma Score**	-	-	-	-	-	-	-	-	50.29	< 0.001	-	-
Mild	20	90.91	2	9.09	0	0.00	0	0.00	-	-	22	5.97
Moderate	61	24.80	106	43.09	46	18.70	33	13.41	-	-	246	66.84
Severe	14	14.00	33	33.00	35	35.00	18	18.00	-	-	100	27.17
**In hospital diagnosis**	-	-	-	-	-	-	-	-	37.60	0.001	-	-
Upper extremity injuries	20	41.67	21	43.75	3	6.25	4	8.33	-	-	48	13.01
Lower extremity injuries	44	22.68	77	39.69	45	23.20	28	14.43	-	-	195	52.85
Both upper and lower extremity injuries	1	5.00	9	45.00	7	35.00	3	15.00	-	-	20	5.42
Polytrauma	12	15.38	28	35.90	24	30.77	14	17.95	-	-	78	21.14
Soft tissues injuries	18	64.29	6	21.43	2	7.14	2	7.14	-	-	28	7.59
**Time before management**	-	-	-	-	-	-	-	-	13.74	0.008	-	-
≤ 1 day	62	34.25	64	35.36	36	19.89	19	10.50	-	-	182	49.32
2–7 days	20	17.24	45	38.79	28	24.14	23	19.83	-	-	116	31.44
8–14 days	3	13.04	11	47.83	6	26.09	3	13.04	-	-	23	6.23
15–30 days	7	23.33	11	36.67	7	23.33	5	16.67	-	-	30	8.13
>30 days	3	16.67	10	55.56	4	22.22	1	5.56	-	-	18	4.88
**Intervention**	-	-	-	-	-	-	-	-	*X*^2^_4_ = 50.94	< 0.001	-	-
Closed reduction+POP	16	40.00	18	45.00	5	12.50	1	2.50	-	-	40	10.84
ORIF	26	16.77	70	45.16	40	25.81	19	12.26	-	-	155	42.01
OREF	6	10.71	15	26.79	21	37.50	14	25.00	-	-	57	15.45
Amputation	1	8.33	2	16.67	4	33.33	5	41.67	-	-	12	3.25
Other	46	43.81	36	34.29	11	10.48	12	11.43	-	-	105	28.46
**Length of Hospital Stay**	-	-	-	-	-	-	-	-	53.07	< 0.001		
0–7 days	63	42.28	54	36.24	21	14.09	11	7.38	-	-	149	40.38
8–14 days	12	21.82	26	47.27	12	21.82	5	9.09	-	-	14	14.91
15–30 days	8	11.27	32	45.07	15	21.13	16	22.54	-	-	71	19.24
>30 days	12	12.90	29	31.18	33	35.48	19	20.43	-	-	94	25.47
**Rehabilitation**	-	-	-	-	-	-	-	-	*W* = 9774.5	< 0.001	-	-
Yes	79	34.05	90	38.79	44	18.97	19	8.19	-	-	232	62.87
No	16	11.76	51	37.50	37	27.21	32	23.53	-	-	137	37.13

ORIF, Open Reduction and Internal Fixation; OREF, Open Reduction and External Fixation; WHODAS, World Health Organization Disability Assessment Schedule.

### Level of disability

Considering the WHODAS score, the minimum score was 0, maximum of 90, with a mean of 22.91. Most participants had mild impairment (38.31%). The overall disability was at 35.86%, combining moderate and severe (Table 3).

**TABLE 3:** Level of functionality Upper Extremity Functional Scale, Lower-Extremity Functional Scale versus WHODAS.

Factors	WHODHAS score									
	No disability (0% – 4%) (*n* = 95)		Mild (5% – 24%) (*n* = 141)		Moderate (25% – 49%) (*n* = 81)		Severe (50%– 100%) (*n* = 51)		Chi-squared	*p*
	*N*	%	*N*	%	*N*	%	*N*	%		
**LEFS**									*X*^2^_4_ = 184.71	< 0.001
Trace functionality (0% – 25%)	0	0.00	2	10.53	4	21.05	13	68.42	-	-
Very poor (26% – 50%)	1	1.56	5	7.81	31	48.44	27	42.19	-	-
Poor (51% – 75%)	8	7.62	57	54.29	34	32.38	6	5.71	-	-
Partial functional (76% – 89%)	17	26.98	43	68.25	3	4.76	0	0.00	-	-
Fully functional (90% – 100%)	69	58.97	34	29.06	9	7.69	5	4.27	-	-
**UEFS**									*X*^2^_4_ = 24.29	0.006
Trace functionality (0% – 25%)	0	0.00	1	16.67	2	33.33	3	50.00	-	-
Very poor (26% – 50%)	0	0.00	1	8.33	6	50.00	5	41.67	-	-
Poor (51% – 75%)	0	0.00	18	75.00	5	20.83	1	4.17	-	-
Partial functional (76% – 89%)	4	23.53	11	64.71	2	11.76	0	0.00	-	-
Fully functional (90% – 100%)	91	29.45	110	35.60	66	21.36	42	13.59	-	-

LEFS, Lower-Extremity Functional Scale; UEFS, Upper Extremity Functional Scale.

Among the 368 patients, the overall disability score of all domains was mild (22.9), the most affected domain was life activities with 26.46 and participation in life with 23.8. All participants were doing well in terms of cognition (6.2) and getting along with people around (5.9) (Table 4). The mean days in the past 30 days that the participants had difficulties in their daily life was 16.5, and the mean days that they could not carry out usual activities or work because of any health condition was 2. We also recorded 2.5 days of reduced usual activities or work because of injury complications. After 2 years of the injury, 134/368 (36.31%) victims of the RTIs were still unable to return to work or perform everyday activities.

**TABLE 4:** Average score of domains and WHODAS 2.0.

WHODAS 2.0 Domains	Score (0 to 100)	Descriptor
Overall disability	22.9	Mild
Cognition	6.2	None
Mobility	18.75	Mild
Self-care	10.7	None
Getting along with people	5.9	None
Life activities	26.46	Mild
Participation	23.8	Mild

### Factors associated with disability

We used a multinomial regression model for the relationship between WHODAS 2.0 scores and associated factors. All associated factors were independent variables of disability: age group, sex, socioeconomic status, KTS, rehabilitation and length of hospital stay. Among these factors, age groups and rehabilitation were significant predictors of disability, with a high odds ratio. The group of > 50 years tends to have 12 times more severe disabilities than the rest. The patients who did not undergo rehabilitation were exposed to severe disability 5.7 times more than the other group (Table 5). We have found that among those younger than 30, the probability of having no disability is 36.9%, mild 49.0%, moderate 9.8% and severe 4.3%. Contrary to a disability, the probability increases in the age group of 30–50 years to > 50 years from 0% to 20% (Figure 2).

**TABLE 5:** Odd Ratio (Multinomial logistic regression/Ref level: No disability).

Factors	Mild	Moderate	Severe
**Age group**			
18–30 years	Ref	Ref	Ref
31–50 years	1.848 (0.986–3.463)	2.605 (1.183–5.738)	2.193 (0.827–5.814)
> 50 years	3.360 (0.986–11.449)	3.778 (0.902–15.824)	12.098 (2.817–51.952)
**Sex**			
Male	Ref	Ref	Ref
Female	1.472 (0.73–2.97)	1.849 (0.825–4.143)	2.369 (0.943–5.953)
**Socio-economic status**			
Category I	Ref	Ref	Ref
Category II	1.711 (0.276–10.617)	0.547 (0.084–3.549)	0.579 (0.082–4.077)
Category III	0.691 (0.118–4.067)	0.314 (0.052–1.887)	0.175 (0.026–1.162)
**Physiotherapy**			
Yes	Ref	Ref	Ref
No	2.088 (1.059–4.118)	3.115 (1.457–6.656)	5.793 (2.430–13.809)
**Length of hospital stay**			
-	1.016 (1.002–1.030)	1.016 (1.011–1.040)	1.029 (1.015–1.044)

**FIGURE 1:**
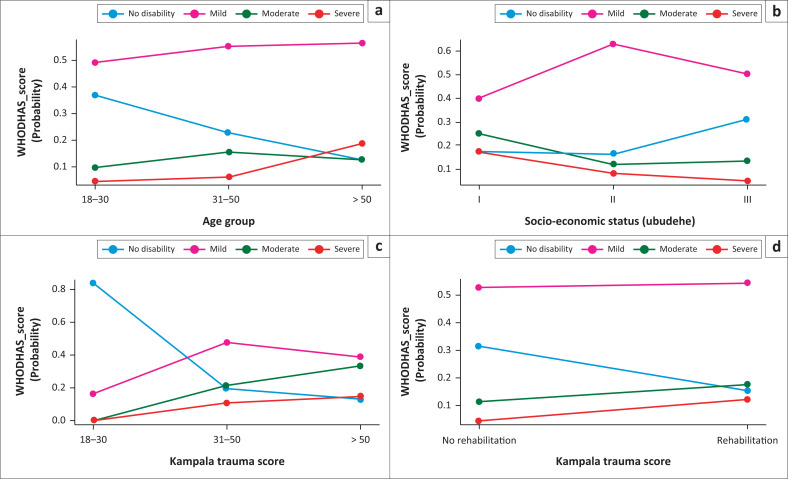
Probability of disability versus age group, socioeconomic status, Kampala trauma score (KTS) and rehabilitation.

The probability of the disability is 20% in Socio-economic Category I, decreasing to 10% for Socio-economic Category II and almost 5% for Socio-economic Category III (Figure 1). The probability of getting the disability increases with the KTS from 0 when there are mild KTS to 20% when the patient has severe KTS. Also, the probability of no disability decreases from 80% for patients with minor injuries to 20% for ones with severe KTS (Figure 1). The patients who had some rehabilitation sessions improved for moderate and severe disabilities, with almost 10% of probability in the non-rehabilitation group (Figure 1).

**FIGURE 2:**
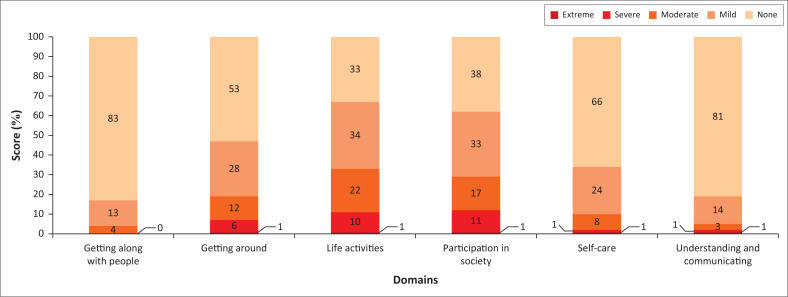
Disability level according to the WHODAS 2.0 Domains.

According to the WHODAS domains, the majority of our participants were not disabled 2 years after a road traffic accident for getting along with people (83%), understanding and communication (81%), self-care (66%), and getting around or mobility (53%). Some WHODAS domains were more affected than others, such as participation in society, where 33% were severely disabled and life activities (28%) (Figure 2).

## Discussion

Our study highlights the magnitude of orthopaedic injuries among RTI victims in Rwanda 2 years after the trauma, where a third of them sustained long-term disabilities. We showed that half of the RTIs in Rwanda are limb trauma, as shown in other studies from LMICs (Access 2018; Mousazadeh et al. 2021). Many factors were associated with disability among the survivors, including demographics, the clinical status of the patient post-injuries, and environmental factors. Age was found to be a contributing factor to the disability level, and one-third of the victims were male. Worldwide RTIs victims are in the working age group (Gheshlaghi & Shari 2020; Gopinath et al. 2015; Marquez 2013), and this was the same finding in our study, where the mean age of our participants was 37.5 years, predominantly in the age group of 31–50 years.

In most studies, especially in sub-Saharan Africa, males are the most involved in RTIs, explained by their high level of mobility (Ingabire et al. 2015; Kim et al. 2016; Lugo et al. 2013; O’Hara et al. 2018). Many studies have shown that the working age group is the most affected by RTIs, which was confirmed in our study, with less than 5% being unemployed (Davey et al. 2019; Gane et al. 2019; Herrera-Escobar et al. 2018). More than half of the participants were in the socioeconomic class category III, composed of people who were gainfully employed or were even employers of labour. These figures explain how accidents are associated with a high rate of movements the victims perform. The leading cause of accidents was motorcycles, followed by a motorcar. In 2012, Rwanda accounted for more than 47,622 motorcycles, more than a half operating as moto-taxi Nickenig Vissoci JR et al. 2020.

Lower limb injuries and polytrauma patients dominated our sample, which is consistent with LMIC studies showing higher rates of lower limb injuries in RTIs (Chauhan et al. 2017; Mahdian et al. 2017). Of these orthopaedic injuries, more than half were managed by operation, either open reduction and internal fixation or external fixation, and the mean hospital stay was 30 days. We observed extended hospital stay for polytrauma patients who required more interventions. Injuries to the upper extremities evaluated by the UEFS had good outcomes compared with those of the lower extremities evaluated by the LEFS. In their systematic review, Rissanen, Berg and Hasselberg (2017) found the same as our findings, where patients with lower limbs do poorly compared with the upper extremities. This was explained by the lower limb injuries’ severity and management (Rissanen et al. 2017). The literature stipulates that the goal of each orthopaedic injury management is to restore the functional outcome, which is achieved by rehabilitation (Heathcote, Wullschleger & Sun 2018). For our study, 37% of the prescribed rehabilitation was not performed after injury management, primarily because of the long distance between their homes and the district hospitals, and financial issues. The same findings were observed in other studies from LMICs where access to rehabilitation ranges from 5% to 59%, and in many countries, rehabilitation centres are lacking (Chatukuta et al. 2022; Joiner et al. 2022; Odland et al. 2022).

Conducting the univariate analysis for both the demographic (age, level of education, residence, occupation) and clinical factors (KTS, diagnosis at admission, time before management, intervention, rehabilitation, UEFS, LEFS), most of the variables independently contributed to the level of disability *p* < 0.05 apart from gender. In their study, Haider et al. (2018) in the United States found almost the same results where demographics (female sex, low education level) play a significant role in the long-term outcome of RTI victims (Haider et al. 2018). Many studies in LMICs share the same picture as our results, with sex, advanced age, rural domicile, and low education level being the independent variables to the disability post-RTI (Glèlè-ahanhanzo et al. 2018; Lin et al. 2013; Locke et al. 2020; Mannocci et al. 2019). The multivariate analysis for this study showed that age group, injury severity score and rehabilitation were highly associated with disability. Pélissier et al. (2017) found that age, injury severity, and post-hospital follow-up are the main predictors of patients’ recovery after 3 years of injury (Pélissier et al. 2017). Glèlè-ahanhanzo et al. (2018) in their study in Benin, identified the same factors with lower limb injuries and rural domicile being among them (Glèlè-ahanhanzo et al. 2018), similar to what Alharbi et al. (2019) have identified in their review.

The prevalence of long-term disabilities post-RTOI is poorly known or under-reported in developing countries. Our study’s overall prevalence of disability was 35.8%, according to the WHODAS categorisation from moderate to extreme disability, but we did not find extreme disability (95% – 100%). These figures are higher than what was reported by WHO report estimating the worldwide disability (15%) and other studies from high-income countries such as European countries ranging from 2.2% to 15.0% (Faux et al. 2015; WHO 2004). Our findings were similar to data from a study conducted in India (Rocha et al. 2016) with 50% of disabilities. Chauhan et al. (2017) in their study from India, found that the prevalence of disability was 13.5% eight months after the injury. Glèlè-ahanhanzo et al. (2018) reported a disability of 10.0% in Benin post-RTIS. Even though the method of disability evaluation differs from study to study, many studies show that the disabilities post RTIs are high in LMICs.

Considering the different domains, our participants were most affected in their participation in life, life activities and mobility, where the level of impairment ranged between 19% and 36%. Our study highlights the magnitude of long-term disabilities following road traffic orthopaedic injuries affecting the victim’s daily activities. Gray et al. (2018) in a study performed in Australia, reported 10% of failure to return to work 2 years after the accident, and Gabbe et al. (2017) in a Cameroun study, reported 83% of the return to work (Gabbe et al. 2017; Gray et al. 2018). Our results are higher than those of these studies, where 36.31% of the RTI victims were still unable to return to work or perform everyday activities after 2 years of the injury. Even for the ones who joined their everyday activities, the mean days in the past 30 days that the participants had difficulties in their daily life was 16.5. The mean days they could not carry out usual activities or work because of any health condition was 2 and 2.5 days of reduced usual activities or work because of injury complications.

## What is new about this study

Our study has determined the prevalence and the level of disability of long-term disability following road traffic orthopaedic injury RTOI in Rwanda. Our findings revealed that the working age is more affected and that a large number lack a rehabilitation follow-up, and a significant number are unable to return to their daily activities after the injuries. This study will serve as the basis for further studies determining the participation in the life of people living with long-term disability following road traffic orthopaedic injuries in Rwanda and their quality of life after 2 years of the accident. These figures will help the stakeholders develop a policy to improve post-RTIs functional outcomes, especially a rehabilitation approach that can quicken post-RTOI functional outcomes.

## Limitations

Our study has shown several limitations, including 2 years between the injury and patient outcome assessment, where some patients were not reached by phone. Using secondary data for baseline also was a challenge. Some missing information in the recording was a limitation that limited the generalisability of our findings. Conducting a cross-sectional study later than a cohort study has also caused missed steps in the patient’s follow-up. We recruited patients involved in the accident in 2019, the year before the coronavirus disease 2019 (COVID-19) pandemic, which limited the regular follow-up of the patients, especially those recruited at the end of 2019. Centre differences also can be a limitation in terms of the homogeneity of the sample size.

## Conclusion

The 2-year prevalence and level of disability because of RTOI in Rwanda is higher than other reported data from high-income countries but comparable to LMICs. Middle-aged and socioeconomically underprivileged persons are the most affected. Disability because of road traffic accidents is related to a greater demand for social and/or healthcare support, problems of accessibility and/or commuting, and significant changes in economic activity. This study has shown that earlier management and rehabilitation are critical for better functional outcomes. We recommend further studies exploring clinical and socioeconomic factors at each patient treatment stage. Furthermore, prospective, and randomised clinical trials can find more about the cause of disabilities and influencing factors.
